# Skeletal Muscle Composition and the Effects of Exercise and/or Prebiotic Fiber in Preventing Diet Related Morbidities

**DOI:** 10.3390/jfmk10020113

**Published:** 2025-03-28

**Authors:** Heiliane de Brito Fontana, Jaqueline Lourdes Rios, John Michaiel, Ruth A. Seerattan, Venus Joumaa, David A. Hart, Raylene A. Reimer, Walter Herzog

**Affiliations:** 1 Human Performance Laboratory, Faculty of Kinesiology, University of Calgary, Calgary, AB T2N 1N4, Canada; heiliane.fontana@ufsc.br (H.d.B.F.); jmichaie@ualberta.ca (J.M.); reimer@ucalgary.ca (R.A.R.); 2 Department of Morphological Sciences, Federal University of Santa Catarina, Florianopolis 88040-900, SC, Brazil; 3 McCaig Institute for Bone and Joint Health, University of Calgary, Calgary, AB T2N 1N4, Canada; 4 Regenerative Medicine Center Utrecht, University Medical Center Utrecht, 3584 CX Utrecht, The Netherlands; 5 Department of Biochemistry and Molecular Biology, University of Calgary, Calgary, AB T2N 1N4, Canada

**Keywords:** obesity, metabolic syndrome, muscle quality, inflammation, dietary intervention, skeletal muscle

## Abstract

**Background:** We established a model of diet-induced obesity in Sprague–Dawley rats that produces, in addition to obesity, metabolic syndrome and musculoskeletal degeneration. Prebiotic fiber and aerobic exercise interventions have been shown to rescue bones and joints from degeneration, but it has yet to be shown if muscle degeneration can also be stopped with these interventions. **Objectives:** This study was aimed at determining if prebiotic fiber supplementation and/or aerobic exercise can prevent muscular alterations in our rat model of obesity. **Methods:** Using a high-fat/high-sucrose (HFS) diet-induced rat model of obesity, 12-week-old male Sprague–Dawley rats were randomly divided into sedentary (HFS, n = 12), exercise (HFS + E, n = 12), prebiotic fiber supplementation (HFS + F, n = 12), or combined intervention (HFS + F + E, n = 12) groups for 12 weeks, with eight chow-fed animals as controls. Muscle triglyceride levels were measured using colorimetric assays, collagen content was assessed histologically, and CD68 immunohistochemistry was performed on the vastus lateralis (VL) and soleus muscles. Group comparisons were conducted using the Kruskal–Wallis test and chi-squared effect statistics (χ2). **Results:** VL triglyceride (χ2 = 10.481, *p* = 0.033) and collagen content in both VL and soleus (χ2 = 23.148, *p* < 0.001 and χ2 = 34.166, *p* < 0.001 respectively) were higher in all HFS-diet intervention groups compared to the chow-fed Control group. Lean body mass did not differ among groups (χ2 = 3.9192, *p* = 0.417). The HFS group exhibited increased total cholesterol and triglyceride levels (χ2 = 11.693, *p* = 0.019; and χ2 = 21.663, *p* < 0.001 respectively) and starkly reduced whole-body insulin sensitivity (χ2 = 18.046, *p* = 0.001) compared to the Control or to the exercise and fiber supplementation groups. **Conclusions:** Despite the effectiveness of aerobic exercise and prebiotic fiber supplementation in preventing the systemic metabolic disturbances induced by the HFS diet, muscular alterations persisted. Prebiotic fiber supplementation led to the highest muscle collagen content, suggesting potential adaptative muscular response to the systemic insult caused by the HFS diet.

## 1. Introduction

Exposure to a high-fat/high-sucrose (HFS) diet is associated with metabolic disturbance that can lead to a variety of co-morbidities and chronic diseases, including musculoskeletal alterations (reviewed in Collins et al. [1]). In the metabolic challenging state induced by HFS diets, skeletal muscles are thought to play a central role in regulating systemic inflammation and metabolic balance [1,2,3]. Muscles are the most metabolically active tissue in humans, accounting for approximately 36–44% of total body mass in healthy young and middle-aged adults, 30% of resting energy expenditure and 80% of insulin-stimulated glucose uptake [4,5].

In contrast to the effects imposed by excessive fat and sugar consumption, physical exercise, specifically aerobic exercise, and prebiotic fiber ingestion have the potential for improving metabolic health [6,7,8,9]. Through aerobic exercise, a complex inter-organ network is created, with important contributions to systemic metabolic health. Aerobic exercise directly affects skeletal muscles, and many of the systemic beneficial effects of exercise are thought to be mediated by skeletal muscles through the production and secretion of myokines through autocrine, paracrine, or endocrine pathways [2,10].

Prebiotic fiber supplementation, such as oligofructose, has beneficial effects that include the stimulation of hormones associated with appetite regulation and the improvement of insulin sensitivity [6,11,12,13]. While exercise and prebiotic fiber supplementation have the potential for counteracting the effects of an HFS diet on the organism, their interactions with an HFS diet and the resulting changes in muscle structure and function remain largely unknown. Research on the effects of prebiotic fiber, and of the metabolites produced by the gut microbiota with fiber fermentation, has focused on interactions with organs, such as the liver, brain, adipose tissue and pancreas [4], with little investigation on the effects of prebiotics on skeletal muscles [14].

Recently, using a Sprague–Dawley rat model, we showed that prebiotic fiber supplementation and aerobic exercise help to maintain the metabolic homeostasis in rats fed an HFS diet, preventing the development of diet-induced insulin resistance and protecting against joint damage that is otherwise observed in this model [15]. However, how muscles react to the interaction between exercise, prebiotic fiber supplementation, and an HFS diet remains unknown.

The purpose of this study was to determine the changes in lean body mass and in vastus lateralis (VL) and soleus muscle composition and macrophage density following a 12-week exposure to a high fat/high sucrose diet, and to assess whether the expected muscle alterations can be prevented if the HFS diet is combined with aerobic exercise and/or prebiotic fiber supplementation.

## 2. Material and Methods

The study design and experimental interventions have been reported elsewhere [15]. The ARRIVE Guidelines 2.0 were used to design and report this study [16,17]. The detailed study protocol was carefully planned as part of the principal investigator’s sabbatical leave and visit at the University of Calgary. The protocol was not registered.

### 2.1. Animals and Protocol

Fifty-six twelve-week-old male CD-Sprague–Dawley rats (original source Charles River Laboratories, Wilmington, MA, USA) were randomized into a chow group (Control, n = 8, diet: 5% of total weight as fat, 47.5% carbohydrates [only 4% from sucrose], 25% protein, 12.5% from fiber and micronutrients, and 10% moisture; Lab Diet 5001, Richmond, IN, USA), a high-fat/high-sucrose diet group (HFS, n = 12, diet: 20% of total weight as fat, 50% sucrose, 20% protein, and 10% from fiber and micronutrients; custom Diet #102412, Dyets Inc., Bethlehem, PA, USA), a high-fat/high-sucrose diet associated with moderate aerobic exercise group (HFS + E, n = 12), a high-fat/high-sucrose diet combined with prebiotic fiber supplementation group (HFS + F, n = 12), or a high-fat/high-sucrose diet combined with moderate aerobic exercise and with prebiotic fiber supplementation group (HFS + F + E, n = 12). Interventions lasted for 12 weeks. The aerobic exercise intervention consisted of a progressive moderate treadmill training program, up to 30 min per day, 5 days a week, as described previously [15]. The prebiotic fiber was supplemented in the HFS diet at a dose of 10% (wt/wt, Orafti P95, BENEO-Orafti SA, Tienen, Belgium) [15]. Animals were individually housed on a 12 h dark/light cycle, and water and food were provided ad libitum. Except for the exercise interventions, there was no environmental enrichment. Block randomization was performed to allocate animals to the Control and treatment groups using the random number generator function in Excel (Microsoft Corporation, Redmond, WA, USA). All 56 rats completed the experiment and were considered in the analyses. The same analytical procedure/test protocol was used for all animals to avoid confounders related to the order/type of measurements and investigators were blind to groups. All experiments were approved by the University of Calgary Life and Environmental Sciences Animal Care Committee, with all methods used being in accordance with the animal welfare regulations and guidelines at the University of Calgary (ethics approval form AC16-0130) and the rules of the Canadian Council on Animal Care (https://ccac.ca/en/guidelines-and-policies/the-guidelines/ accessed on 1 April 2020). Results associated with joint and systemic health that emerged from this study have been previously published [15].

### 2.2. Body Composition and VL and Soleus Muscle Masses

Three days after completing the 12-week intervention protocol, rats were lightly anaesthetized with isoflurane and body composition (body mass, body fat, and lean body mass) was measured using Dual X-ray absorptiometry (DXA) with software for small animals (Hologic ODR 4500; Hologic Inc., Bedford, MA, USA). Prior to each testing session, the DXA machine was calibrated using phantoms representative of hard (bone) and soft tissue (muscles, fat) densities. The mean of three repeat scans for each animal was used for the analysis.

At sacrifice, the VL and soleus muscles were harvested and the whole muscle mass was measured (g). Muscles were immediately flash frozen in isopentane cooled in liquid nitrogen and stored at −80 °C.

### 2.3. Muscle Collagen Content

One slice from the mid-belly of the soleus and the VL tissue was removed with a scalpel and mounted in O.C.T. compound (Tissue-Tek^®^ optimal cutting temperature mounting medium) and subsequently 10 µm serial cross-sections were cut using a cryostat at −20 °C. Sections were mounted on Superfrost™ Plus glass slides and stored at −25 °C before staining (Picrosirius Red staining and CD68 immunostaining). For Picrosirius Red staining, slides were oven dried at 40 °C for 7 days and then fixed overnight in a 10% neutral buffered formalin solution. Slides were immersed in Bouin’s fixative solution for 1 h at 60 °C and washed in distilled water. The picrosirius red solution was made by adding 0.1 g of Sirius Red stain (Sigma-Aldrich, Oakville, ON, Canada), to 100 mL of a saturated aqueous picric acid (1.3% in water, Sigma-Aldrich). Sections were stained in this solution for 1 h at room temperature.

Slides were rinsed in 0.5% acetic acid, dehydrated in three changes of 100% ethanol, cleared in xylene, and mounted with DPX mounting media. Sections were imaged using 10× objective on an Olympus BX53 light microscope (Olympus, Tokyo, Japan) and analyzed using ImageJ (version 1.53k).

The intensity of the stain was averaged across each muscle section (30–50 frames/animal). Data from Picrosirius Red are presented as a percentage of the muscle cross-sectional area.

### 2.4. Muscle Triglyceride Content

A second slice from the mid-belly of the soleus and the VL tissue was removed with a scalpel. The slices were freeze-dried for 48 h and then used to quantify the triglycerides content in the muscles using a triglyceride colorimetric assay (Triglyceride (GPO) (Liquid) Reagent Set, Pointe Scientific Inc., Canton, MI, USA])according to the manufacturer’s instructions.

Data from colorimetric assays are presented as a percentage per gram of dry muscle.

### 2.5. Muscle Macrophage Density

Immunostaining for CD68+ cells was performed to quantify macrophage density in VL and soleus muscles, as described previously [18]. Briefly, slides were fixed in cooled (4 °C) 100% acetone. Sections were then blocked with 3% hydrogen peroxide in PBS, followed by 2.5% normal horse serum blocking (Vector Laboratories, Burlingame, CA, USA). Sections were then incubated with the primary antibody, ED1 mouse anti-rat CD-68+, a marker for macrophages (AbD Serotec, Raleigh, NC, USA) at 1:200 dilution overnight at 4 °C. The secondary antibody, ImmPRESS anti-mouse IgG (Vector Laboratories, Burlingame, CA, USA) was applied for 30 min at room temperature. Sections were incubated in a Cyanine-3 fluorophore using the TSA-Cy3 system (Tyramide Signal Amplification TSA Kit: PerkinElmer, Waltham, MA, USA) for 20 min at 1:500. Then, 4′6-diamidino-2-phenoylindole (DAPI) was applied to visualize nuclei, and sections were mounted with Vectashield aqueous mounting media (Vector Laboratories). Ten random images per VL and nine per soleus cross-section were obtained using the 20× objective on an Olympus Fluoview FV1000 confocal microscope (Olympus, Tokyo, Japan), a QImaging^®^ Retiga 2000R camera, and QCapture Software version 3.1 (QImaging, Surrey, BC, Canada). The CD 68+ cells were counted by a blinded investigator, and the number of CD68+ cells was averaged across each muscle image frame. Cells were considered positive if simultaneously stained for DAPI and Cyanine-3. Data from CD68 staining are presented as positive cells per 0.25 mm^2^.

### 2.6. Blood Glucose and Insulin

One day after the end of the intervention period, and following 16 h of food deprivation, rats were given an oral gavage of 2 g/kg glucose. Blood was collected at 0, 15-, 30-, 60-, and 120-min post gavage via tail nick in a chilled tube for insulin analysis. Blood glucose was measured immediately with a blood glucose meter (OneTouch Verio and Blood Glucose Monitoring System, LifeScan, Zug, Switzerland). Whole body insulin sensitivity was determined using proxy measures from the glucose tolerance tests (composite insulin sensitivity index—CISI). Data are presented as fold change from the chow-fed Control group.

### 2.7. Blood Lipid Profile

One week prior to the start of the 12-week intervention protocol (acclimatization week) and following 16 h of food deprivation, blood was collected via tail nick to evaluate the baseline lipid profile. Four days after completing the 12-week intervention protocol and following 16 h of food deprivation, rats were anaesthetized with isoflurane and cardiac blood samples were collected. Serum was analyzed for lipid profiles (total cholesterol, LDL-cholesterol, HDL-cholesterol, and triglycerides) using colorimetric assays (Calgary Lab Service, Calgary, AB, Canada). Data are presented as fold changes from baseline.

### 2.8. Statistical Analysis

Descriptive statistics are shown as a mean and standard deviation in the presence of a normal distribution or as a median and interquartile range, as indicated in the text. Non-parametric Kruskal–Wallis testing with the Dunn test for multiple comparisons was used to determine differences between the five animal groups for all variables. For the multiple comparisons, *p*-values were adjusted using the Benjamini–Hochberg method. Analyses were performed using R software (version 4.0.5 R Foundation for Statistical Computing, Vienna, Austria). Kruskal–Wallis chi-squared statistics (χ2) are reported. Data were considered statistically significant at *p* < 0.05, two-tailed test.

## 3. Results

**Figure 1:**
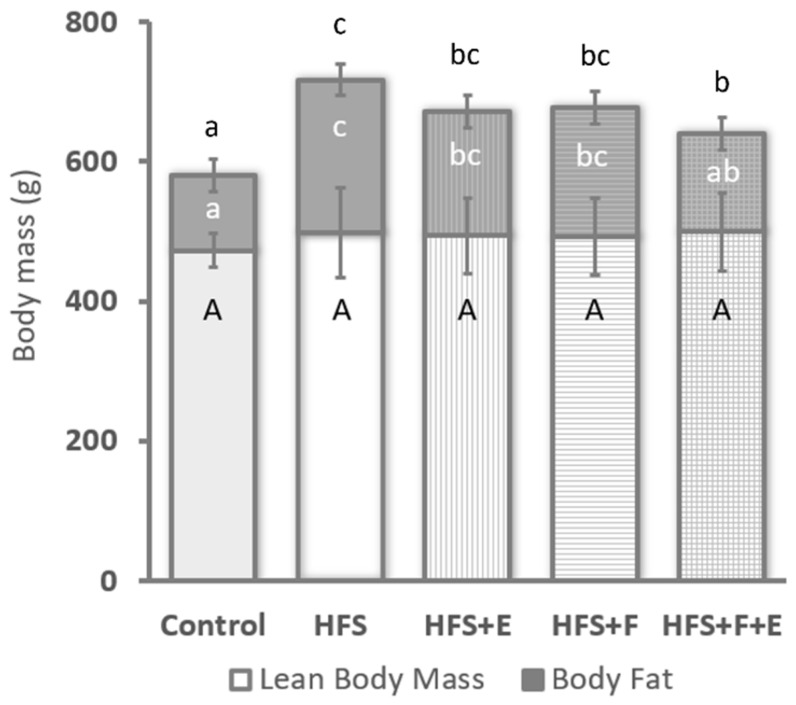
Mean and SD of body fat and lean body mass at the end of the experimental protocol. Equal letters indicate similar values between conditions while different letters indicate a statistically significant difference at *p* < 0.05. Capital letters used for comparing lean body mass and small letters used for comparing body mass (black small letters) and body fat (white letters). n = 12; except Control, n = 8.

Body mass differed across groups at the end of the intervention period (χ2 = 19.8, df = 4, *p* < 0.001), with rats fed a standard chow diet being lighter than rats from any of the groups that received the HFS diet (*p* < 0.05). Additionally, rats in the HFS + F + E group were lighter than those in the HFS group. No differences in lean body mass were observed across groups (χ2 = 3.92, df = 4, *p*-value = 0.42, Figure 1, but a main effect for body fat was detected (χ2 = 21.0 df = 4, *p* < 0.001) (Figure 1). Except for the HFS + F + E group, rats fed the HFS diet (HFS, HFS + E and HFS + F) had more body fat than rats fed a standard chow control diet (*p* < 0.05). Body fat in rats from the HFS + F + E group was similar to that of the chow-fed rats, HFS + E rats, and HFS + F rats, but was lower than in rats that received the HFS diet alone.

**Figure 2:**
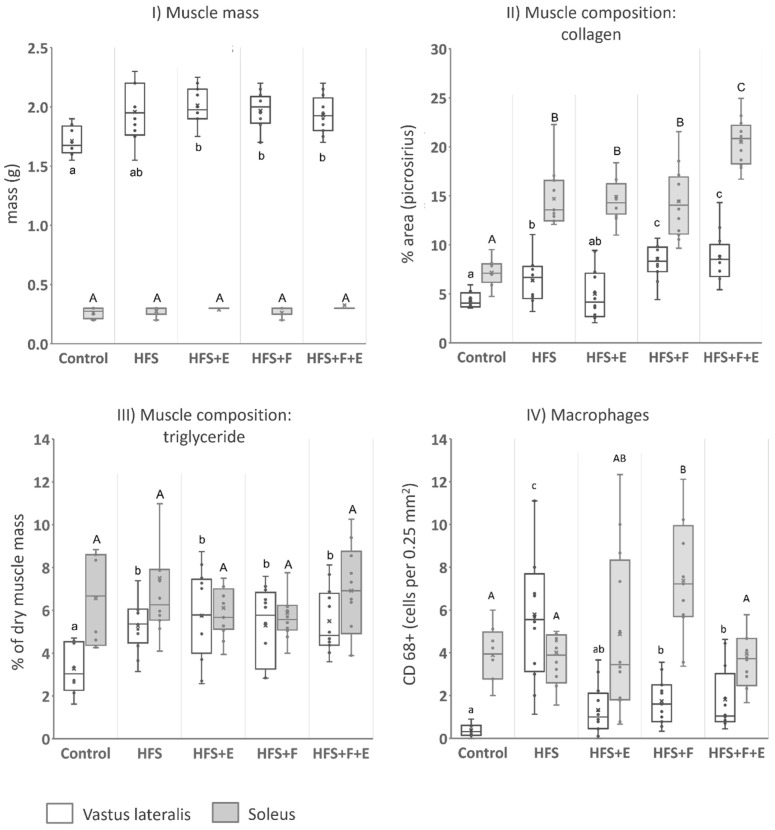
Vastus lateralis (white) and soleus (shaded) mass, composition, and macrophage density represented in box plots (median, mean, and interquartile interval) after 12 weeks of a high fat/high sucrose diet alone and combined with prebiotic fiber and/or exercise interventions (HFS: high fat/high sucrose diet; F: prebiotic fiber; E: aerobic exercise). (**I**) Muscle mass, (**II**) collagen content, (**III**) triglyceride content, and (**IV**) macrophage density. Equal letters represent similar values across groups, while different letters indicate a statistically significant difference at *p* < 0.05. Capital letters refer to comparisons regarding the soleus muscle and small letters refer to comparisons for the VL. n = 12; except Control, n = 8.

VL mass in rats that received the HFS diet combined with prebiotic fiber supplementation and/or exercise was greater than in the Control rats (main effect across groups: χ2 = 13.1, df = 4, *p* = 0.011 (Figure 2I). For the soleus muscle, no significant differences in muscle mass were observed (main effect across groups: χ2 = 8.2, df = 4, *p* = 0.084)).

Both the VL and soleus muscles of rats fed the HFS diet (HFS, HFS + F, HFS + E and the HFS + F + E) exhibited greater collagen content compared to those from the chow-fed Control group (main effect across groups: χ2 = 23.1, df = 4, *p* < 0.001 for VL and χ2 = 34.2, df = 4, *p* < 0.001 for soleus). A higher collagen content was observed in the soleus of the HFS + F + E group compared to the group that received the HFS diet alone, and in VL, it was higher in both the HFS + F and HFS + F + E groups compared to HFS alone (Figure 2II). Furthermore, exercise (HFS + E) mitigated the increase in collagen content observed in the VL of the group that received the HFS diet alone.

Rats in the HFS diet group showed increased intramuscular triglyceride content in the VL muscle compared to the Control group values (main effect across groups: χ2 = 10.5, df = 4, *p* = 0.03), while no differences were observed across groups for the soleus muscle (main effect across groups: χ2 = 2.5, df = 4, *p* = 0.64). Moderate aerobic exercise, prebiotic fiber supplementation, or their combination did not prevent the observed increases in VL triglyceride content in the groups fed the HFS diet (Figure 2III).

The concentration of immunoreactive cells for CD68 was highest in the VL for the HFS group, followed by the HFS + F and HFS + F + E groups. The Control and the HFS + E groups presented the lowest concentration of CD68 immunoreactive cells. In the soleus, aside from the observed increase in the HFS + F group, concentrations of immunoreactive cells were similar between the chow-fed Control and intervention groups (Figure 2IV).

**Figure 3:**
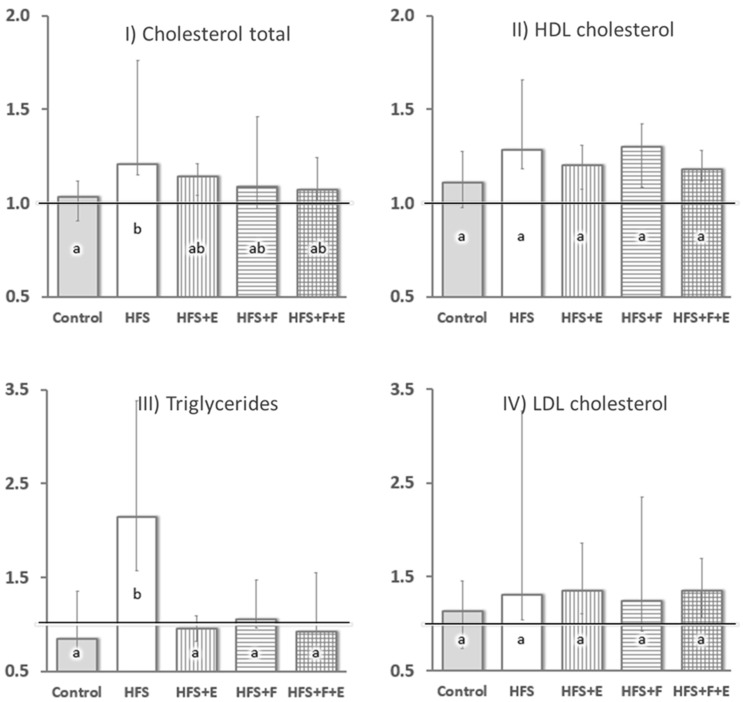
Serum lipid profile expressed as fold change (Y-axis) from baseline measurement (horizontal line). (**I**) Cholesterol Total, (**II**) HDL Cholesterol, (**III**) Triglycerides, (**IV**) LDL Cholesterol. Equal letters represent similar changes from baseline across groups while different letters indicate a statistically significant difference at *p* < 0.05. HFS: high fat/high sucrose diet; F: prebiotic fiber; E: aerobic exercise. Data are presented as median and interquartile interval. n = 12; except Control, n = 8.

At sacrifice, there was a marked difference in insulin sensitivity across groups (χ2 = 18.0, df = 4, *p* = 0.001): HFS group rats had less than half of the whole-body insulin sensitivity of rats in the other groups. No differences between the Control, HFS + E, HFS + F, and HFS + F + E groups were observed. Additionally, the HFS diet led to increased serum total cholesterol (χ2 = 11.7, df = 4, *p* = 0.019) and triglyceride levels (χ2 = 21.7, df = 4, *p* < 0.001) compared to rats fed the chow diet (Control group, Figure 3). Prebiotic fiber supplementation, exercise, and the combination of the two prevented increases in serum triglyceride levels (Figure 3) and mitigated the increase in total cholesterol observed with the HFS diet alone.

## 4. Discussion

Our study was aimed at determining the effects of an HFS diet alone and in combination with moderate aerobic exercise and/or prebiotic fiber supplementation on the VL and soleus muscles in rats. Sprague–Dawley rats were used as they have been shown previously to be an excellent model for diet-induced obesity and associated degeneration of muscles, bones and joints in the presence of an HFS diet (e.g., [1]). Using aerobic exercise and prebiotic fiber supplementation, such degeneration of the bones and joints could largely be prevented [15], but the effect of these interventions on muscles has not been systematically explored in this pre-clinical model. In the current study, we observed significant alterations in muscle composition across all intervention groups when compared to control group muscles, regardless of exercise or fiber supplementation. Interestingly, neither exercise nor prebiotic fiber supplementation prevented these alterations. This result differs from previous findings regarding joint, cartilage, and subchondral bone, where combining the HFS diet with prebiotic fiber supplementation and/or moderate aerobic exercise prevented the joint damage that was otherwise observed in this model with the HFS diet alone [15]. Our current investigation on muscles confirms that this protective effect on joints occurs in the presence of significant changes in muscle composition compared to the chow-fed Control rats.

We evaluated lean body mass using DXA. While important changes in muscle composition were observed across groups (Figure 2), lean body mass remained similar to the chow-fed Control rats (Figure 1). The discrepancy between lean body mass and muscle composition highlights the importance of investigating muscle composition—often referred to as muscle quality—when analyzing muscle mass. Muscle mass estimates by DXA scan have been shown to underestimate sarcopenia and cannot be used in isolation to accurately infer contractile muscle mass [19]. Notably, the trends observed in VL and soleus muscle mass after dissection (Figure 2I) were similar to our findings for muscle triglyceride content in both muscles (Figure 2III).

VL muscle exhibited increased triglyceride content and inflammatory cell infiltration in response to the HFS diet, while the soleus muscle appeared relatively protected from these changes. Our results for the soleus muscle agree with the findings reported by Collins et al. [20], who showed that soleus fat content was conserved after 12 and 28 weeks of a high-fat/high-sucrose diet compared to chow-fed control animals. The lack of fat infiltration in the soleus muscle in response to the HFS diet might be related to its oxidative capacity, with a greater concentration of type I fibers, and consequently a higher concentration of mitochondria, denser capillarity, and lipoprotein lipase activity than VL [1,21,22,23]. Fiber types with high mitochondrial oxidative activity have an increased capacity for uptake and oxidation of fatty acid—the building blocks of fat [22,24,25]. While previous studies did not observe obesity-related differences in fat accumulation between type I and type II fibers in the VL in humans [22] and in the gastrocnemius of rats [26], the effect of obesity across muscles of different fiber type composition has not been systematically investigated.

The HFS diet, either in isolation or combined with aerobic exercise and/or prebiotic fiber supplementation, led to changes in VL triglyceride composition that approximated the content of the soleus. This finding agrees with the variation in the metabolic profile and insulin-mediated glucose uptake for different muscle fiber types [1,20]. While a detrimental effect on force output capacity with muscle fat infiltration can be expected [27], the increase in triglyceride content in the predominantly fast-twitch-fibered VL might reflect an adaptation towards increased fat oxidation and fat reserve capacity, to minimize the metabolic disturbance caused by the HFS diet on the other tissues [1,23,28]. Triglycerides stored in striated muscle cells vary with diet, muscle fiber type, and physical fitness level. Interestingly, previous studies showed that, both exercise training and high fat intake may lead to adaptations in lipid metabolism in skeletal muscle that result in greater storage of fat in the muscle [28,29,30].

Of note is the dissociation between systemic insulin resistance and the intramuscular fat or immunoreactive CD68+ cells observed across groups. Skeletal muscle is a highly plastic tissue that plays a central role in human health and disease, with previous studies suggesting that skeletal muscle inflammation, and the associated changes in muscle composition that occur with obesity, are important contributors to insulin resistance [1,3,31]. Our results seem to indicate that intramuscular triglyceride content in VL does not determine the development of systemic insulin resistance, nor should it be used as a marker of decreased insulin sensitivity in obesity, as previously suggested [32]. While exercise and/or prebiotic fiber were able to fully prevent the decrease in insulin sensitivity that is otherwise observed when the HFS diet is administered alone, clear changes in muscle composition were observed in all groups that received the HFS diet, regardless of exercise or fiber supplementation.

Prebiotic fiber supplementation alone did not prevent the increase in VL triglyceride content with the HFS diet, as one might have expected, but contributed to a further increase in collagen content when compared to HFS alone (Figure 3II). Little is known of the effects of prebiotics on muscles, especially when used as a supplement to an HFS diet. The oligofructose prebiotic fiber supplementation constitutes a non-digestible plant-derived carbohydrate that is fermented by the gut microbiota and leads to the production of short-chain fatty acids in the colon. Locally, short-chain fatty acids can suppress the growth of gut pathogens and influence intestinal motility [11]. With regards to systemic effects, after their rapid absorption by the colonic mucosa, short-chain fatty acids can contribute towards the energy requirements of the host and have well-established effects on whole-body energy homeostasis [4,33].

There is preliminary evidence pointing to a decrease in muscle fibrosis with dietary fatty acid supplementation in pigs [34], and previous studies showed that the consumption of a diet rich in fatty acids results in adaptations in several skeletal muscle proteins that are similar to those observed with exercise training [28]. To our knowledge, this is the first investigation on muscles concerning the interaction between prebiotic fiber supplementation and an obesogenic diet. It is possible that changes in muscle metabolic flexibility, that result from the exposure to the HFS diet [28,35], moderate the effects of prebiotic fiber and/or fatty acid supplementation. Skeletal muscles in aerobically trained individuals are characterized by an increased capacity to oxidize fatty acids, and muscles of lean individuals have the ability to shift to increased levels of fatty acid oxidation in response to a high-fat diet [28,35,36]. By contrast, in obese individuals, some rigidity in increasing fatty acid oxidation in response to high fat intake has been observed [37]. It is possible that the effects of fatty acids and prebiotic fiber supplementations on muscle fibrosis observed in combination with a regular diet change when combined with an HFS diet.

Studies in which fatty acid supplementation and aerobic exercise training in individuals with obesity have been compared, suggest that exercise is a more effective tool to improve fatty acid oxidation and overcome the lipid metabolic inflexibility that seems to be at the core of the metabolic syndrome [28]. Recently, van den Hoek et al. [32] verified that neither 20 weeks of exercise nor a change to a low-fat diet could reverse the 45% increase in collagen content in mice quadriceps muscle after 30 weeks of exposure to a high-fat diet, when compared to a chow-fed control group. By contrast, the authors reported that the exercise and dietary change, either in isolation or combined, decreased substantially the 90% increase in intramuscular triglycerides that was observed after 30 weeks of a high-fat diet [35]. In the current study—except for the non-significant mitigation in VL collagen proliferation in the group that combined the HFS diet with exercise compared to the HFS alone group (Figure 2II)—exercise and prebiotic fiber supplementation on top of the HFS resulted in similar or greater changes in muscle composition than the HFS diet alone. While in our study, the interactions between diet, exercise, and prebiotic supplementation were tested with the interventions starting concomitantly, in the study by van den Hoek et al. [32], the metabolic challenge posed by the high fat diet was substituted by a low-fat diet and/or exercise—a substitution that might be necessary to cause a decrease in intramuscular triglycerides.

The HFS-diet experimental model used in this study induces obesity and reliably produces systemic inflammation and metabolic alterations with increased insulin resistance and dyslipidemia in rats [1,15,38]. These metabolic alterations are typically associated with an increased risk of premature mortality and are related to the development of many chronic diseases, including osteoarthritis-like damage to the knee and shoulder joints [15,38]. The mechanism and clinical significance of the changes in muscle composition in the groups fed an HFS diet, especially the additional collagen content increase observed when the HFS diet was combined with prebiotic fiber, require further investigation. Muscular fibrosis may contribute to the decline in endurance and coordination observed with prolonged exposure to a low-quality diet [7]. These changes can contribute to a vicious cycle of muscle degeneration, reduced physical function, and sedentarism, aggravating the metabolic disturbance.

In the absence of other studies investigating the combined effects of dietary fiber supplementation and exercise in the presence of an HFS diet on muscle health, it is difficult to anticipate how the results of this study might translate to other species than Sprague–Dawley rats. Research on rodents has been shown that diets high in fat, and or high in fat and sucrose, produce similar changes to those observed in humans and have been recommended as excellent models of human metabolic disturbance associated with obesity (e.g., [39,40]) However, most studies on nutritional and exercise interventions in the context of obesity do not specifically examine degeneration of the musculoskeletal system. Notably, a six-month oligofructose intervention in humans with obesity and mild to moderate knee osteoarthritis showed promising effects on pain reduction and decreased use of pain medication [41]. Similarly, exercise interventions in humans with knee osteoarthritis have been associated with improvements in function and reductions in pain [42]. These results suggest, but by no means prove, that models of obesity and dietary and exercise interventions in rodents have similar effects on musculoskeletal health in humans. However, rodent models have the advantage over human research that mechanistic pathways using invasive methods can be studied under precisely controlled conditions, and that musculoskeletal changes tend to occur over much shorter time periods than in humans due to the vastly increased metabolic rates of mice and rats compared to humans.

Future studies should investigate changes in contractile muscle proteins and properties for the interaction between an HFS diet, exercise, and prebiotic fiber supplementation. Additionally, further investigation of macrophage density is necessary, as we obtained highly variable results, especially with regards to the soleus muscle. This result might be related to the fact that CD68+ immunoreactivity has also been observed in non-myeloid cells with the intensity of CD68+ staining of primary fibroblasts and endothelial cells being comparable to that of macrophages [43]. The observed concentration of CD68+ cells for each group, thus, should be interpreted with caution.

## 5. Conclusions

Moderate aerobic exercise and prebiotic fiber supplementation were effective in counteracting the metabolic disturbance posed by the HFS diet, with insulin sensitivity and serum lipid profiles maintained at control levels. However, alterations in muscle composition were observed in all groups fed the HFS diet, regardless of exercise or fiber supplementation. It is possible that these alterations are associated with muscular mechanisms that emerge as an attempt to cope with the disturbance created by the HFS diet. Future studies are needed to analyze the functional implications of the increased collagen content associated with prebiotic fiber supplementation.

## Data Availability

Data will be made available upon request to the corresponding author.
